# Planning ahead in public health? A qualitative study of the time horizons used in public health decision-making

**DOI:** 10.1186/1471-2458-8-415

**Published:** 2008-12-18

**Authors:** David C Taylor-Robinson, Beth Milton, Ffion Lloyd-Williams, Martin O'Flaherty, Simon Capewell

**Affiliations:** 1 Division of Public Health, The Whelan Building, Quadrangle, Liverpool, L69 3GB, UK

## Abstract

**Background:**

In order to better understand factors that influence decisions for public health, we undertook a qualitative study to explore issues relating to the time horizons used in decision-making.

**Methods:**

Qualitative study using semi-structured interviews. 33 individuals involved in the decision making process around coronary heart disease were purposively sampled from the UK National Health Service (national, regional and local levels), academia and voluntary organizations. Analysis was based on the framework method using N-VIVO software. Interviews were transcribed, coded and emergent themes identified.

**Results:**

Many participants suggested that the timescales for public health decision-making are too short. Commissioners and some practitioners working at the national level particularly felt constrained in terms of planning for the long-term. Furthermore respondents felt that longer term planning was needed to address the wider determinants of health and to achieve societal level changes. Three prominent 'systems' issues were identified as important drivers of short term thinking: the need to demonstrate impact within the 4 year political cycle; the requirement to 'balance the books' within the annual commissioning cycle and the disruption caused by frequent re-organisations within the health service. In addition respondents suggested that the tools and evidence base for longer term planning were not well established.

**Conclusion:**

Many public health decision and policy makers feel that the timescales for decision-making are too short. Substantial systemic barriers to longer-term planning exist. Policy makers need to look beyond short-term targets and budget cycles to secure investment for long-term improvement in public health.

## Background

Much effort and resource goes into developing the broad research base that should ideally inform important healthcare policy decisions[1]. However, when compared to the resources devoted to biomedical research, relatively little work has been undertaken to understand the factors that influence the decision making process in practice. A better understanding of the decision making process in public health is needed. This is particularly relevant at the local level in the UK, where there is a drive to improve the quality of commissioning of health services, and Primary Care Trusts are expected to become world-class commissioning organizations[2,3]. In order to do this public health practitioners need to be advocates for change and to be able to marshal evidence in such a way that it can be used in making the case for change with other colleagues and stakeholders. Commissioners and policy makers exert an important influence on population health through the decisions they make regarding coronary heart disease (CHD), cancers and other diseases. Understanding these processes may highlight policy levers for those wishing to influence health policy to improve population health. Previous qualitative research has emphasised the importance of researchers understanding the 'real-life' constraints around the policy process [4]. In addition research has suggested that the use of evidence in decision making is tempered by shifting timescales and financial constraints in the UK National Health Service (NHS) [5].

Using CHD as an exemplar, we therefore designed a qualitative study of NHS policy makers, commissioners, and other decision makers within the CHD planning arena, to explore factors that influence the policy and decision making process. We specifically intended to explore the issues relating to the time-scales within which public health decisions are being made, to explore the notion of a culture of short termism within the NHS [6]. This research was part of a larger programme designed to inform the development of the IMPACT CHD policy tool [7], including exploring the role of research in the decision-making process, key policy issues around CHD, and the role of decision-support systems for CHD policy[8].

## Methods

### Participants and setting

The sampling frame comprised 58 individuals involved in the policy and decision-making process around CHD in a range of organisations in England, Scotland and Wales. Two distinct strategies were used to generate this diverse pool of participants: Firstly, a list of known individuals involved in the policy and decision-making process was drawn up on the basis of existing professional networks. Secondly, a purposive sampling strategy explicitly sought to include individuals from a wide range of areas across England, Scotland and Wales, and from any organisational types that were under-represented in the initial list of known policy/decision makers' organisations. These lists were then combined to generate the final sampling frame. A recruitment letter was sent to every person on the list – this gave background details and invited the recipient to participate in the study. In addition, we asked interviewees to identify any other important CHD decision or policy makers – we then sought to recruit individuals from any groups not represented in our original sampling frame.

### Interviews

Data were collected by individual semi-structured interviews, conducted at a time and venue convenient for the participant. Prior to the interview, participants received an introductory letter, which provided further information about the consultation process. The interviews opened with general questions about the participant's role, organisation and professional background. Non-directive interview questions then explored key policy concerns in relation to CHD and current decision-making practices. We explicitly explored issues relating to the timeframes used for decision-making, and the factors that influence this process. The interviews were conducted by DTR and BM. Data were digitally recorded and then subsequently transcribed verbatim.

### Analysis

Data were analysed using NVivo software for qualitative data analysis (version 7). The analysis used techniques drawn from the framework method of qualitative data analysis [9,10]. DTR and BM carried out a familiarisation analysis, and identified a thematic framework. This thematic framework drew on both a priori issues and also on concepts that emerged from the data during the data collection and familiarisation analysis stages. The thematic framework was converted into a series of codes to be applied to the data. Data from all of the transcripts were systematically coded, charted and mapped. The analysis then sought to identify associations between themes and to carry out an in-depth exploration of the emergent findings.

### Validation

Before the whole dataset was indexed by DTR and BM, a third researcher (FLW) independently coded a sub-sample of transcripts and then compared coding, as a check to ensure high levels of inter-researcher consistency in analysis.

### Ethics

We sought advice regarding ethical approval from the appropriate committee and were advised that the project did not require formal review under the terms of guidance for research ethics committees in the UK. All interviewees gave informed consent to take part in the study.

## Results

Of the 58 people included in the sampling frame, 27 initially agreed to take part in the consultation. In addition, 3 people nominated other participants from within their organisations or their professional networks, all of whom agreed to take part.

As the data collection process progressed, it became clear that we also needed to speak to Directors of Finance and those involved directly in commissioning CHD services. Three additional participants in these professional roles were recruited to supplement the existing sample, giving a total of 33 participants (table 1).

**Table 1:** List of participants

Type of Organisation	Country	Number
National level decision makers from Departments of Health	England and Scotland	6

Regional level decision makers from Strategic Health Authorities	England, Wales and Scotland	4

Directors of Public Health or Consultants in Public Health at local level (Primary Care Trusts)	England, Wales and Scotland	9

Directors of Finance	England	2

Commissioners	England	1

Physicians	England	2

Senior Academics with direct experience of policy making	England and Scotland	5

Voluntary organisations	England	4

### Timescales for Decision Making

Policy makers varied in their responses with regard to the usual timescales for decision-making. The most common timescales cited tended to be relatively short, at around one to three years. However, many decision makers seemed to feel that the timescales were too short. Decision makers at the local level, and some of those at the national level tended to report short timeframes. There was the impression that the way in which the 'system' was set up necessitated this short-termism. This was especially the case with regard to the commissioning and funding cycle within the NHS, especially at the local level.

"The trouble is the NHS thinks in yearly decisions. In policy terms, 3 years is the length of time people tend to think. The NHS operating framework is a 3 yearly cycle and the comprehensive spend round is 3 yearly."

Decision maker – National Level

"I think certainly the NHS has been guilty and it is not alone in this, of tending to plan on too-short a timescale."

Public Health Consultant – Regional Level

"Unfortunately the NHS thinks in quarters at the moment so a big step forward would be if we could get commissioners to think in terms of 3 – 5 years"

Director of Public Health – Local level

For those working around CHD policy in voluntary organisations (outside of the NHS), responses tended to indicate that there was more scope for longer term planning and horizon-gazing.

"For me it would be a minimum 5 to 10 years and possibly longer"

Director – Voluntary Organisation

A number of respondents suggested that the timescale for decisions depended on the issue at stake, and the nature of the organisation.

"*There are different time cycles; that's the obvious answer to that. Things that end in policy are usually in that 2 to 4,5 year cycle."*

Academic

### Importance of longer time-horizons

Table 2 outlines the reasons that respondents gave around the importance for long term planning in public health. Respondents felt that more long term planning was needed if public health policies are going to influence the wider determinants of health, and to facilitate broader societal changes.

**Table 2:** Drivers for longer-term thinking

○ Action on determinants of health
○ Facilities planning
○ Regeneration and population health improvement
○ Capital planning
○ Workforce development
○ Systems change
○ Societal change

"But I think we do need to actually have longer-term strategies as well, in terms of 10 – 20 years. So for example we've just produced a health and improvement strategy in the city, which is a 10 year health improvement strategy, and the city's regeneration strategy is a 15 year strategy. To bring about the change in society that's required you have to really have a 10 year plan rather than a 3 year plan."

Public Health Consultant – Local level

Respondents also suggested that longer term planning was needed in public health in the context of human resources and capacity planning, and estates management.

"When you're building or closing down big facilities you're working on a much longer timeframe"

Public Health Consultant – Regional Level

### Reasons for short-term planning in public health

Respondents gave a range of possible reasons for short timeframes in public health decision-making. These are outlined in Table 3. The two main themes relate to organisational systems, and the difficulty of long term forecasting.

**Table 3:** Drivers for short-term planning

**Organisational systems**

○ Politicians and public perceived as thinking in terms of short term electoral payback
○ Policies perceived to have limited shelf life
○ Commissioning framework is short term
○ Evidence-based policy making leads to a focus on short term interventions evaluated by randomised control trials (RCTs)
○ Disruption caused by reorganisations
○ System set up (the NHS and the government)
○ European legislation

**Difficulty of long term forecasting**

○ Service reorganisations
○ Perceived poor quality evidence for long-term intervention
○ Research not produced in a timely fashion to inform decisions
○ Focus on short term targets and deadlines
○ Accuracy of forecasting limited
○ Uncertainty of effects of major long term processes like climate change and global food shortages on public health
○ Public heath professionals perceived to have limited influence on long term planning process
○ Lack of long-term perspective think tank

### Organisational systems

Two of the most common suggestions were that the political and financial cycles were intrinsically short term. A number felt that the 4 yearly political electoral cycle was the backdrop against which all planning was made, and that this naturally led to time constraints in planning within the health service.

"Its pretty short, partly because the overall way Government works it doesn't support longer term planning really."

Decision maker – National Level

"I think if you're talking about high level policy makers, Ministers or top Civil Servants then the time frame is relatively short a year or six months, so if you can't do the research or do the findings within a year then they're not going to be interested."

Voluntary Organisation

"Politicians will probably have a shorter view point because they're thinking about the next election."

Academic

In addition there was the suggestion that there is a natural turnover of policies, and that they need to be reviewed every few years, as people move on in roles, and as policy directions shift and evolve.

"There's something about policies that sort of have a limited shelf life, and then stuff happens and even though they seem quite fresh in the first few years...people change jobs, politicians change and it runs out of steam... and after about 5 years people get bored and move on. I've not seen many strategies over 10 years old that are actually still healthy and you really have to think hard to think of longer strategies, can you think of any?"

Decision maker – National Level

Respondents felt that the commissioning and budget cycles within the NHS are geared up to achieve financial balance over relatively short time periods.

"We an never get away from the fact that we have to do things that do show an impact fairly quickly....There's an obsession up until this year that means you must not make a surplus; success is breaking even, not more not less. That leads you to short term spending."

Decision maker – Regional Level

Many participants felt that the frequent reorganisations within the health service were felt to be particularly disruptive in terms of long term planning.

"The unfortunately fact is that most organisations in commissioning terms haven't lasted more than 5 years."

Public Health Consultant – Regional Level

"...we want to measure to say we've made a difference because in two years' time we'll probably be reorganised."

Director of Finance – Local level

### Difficulties with long term forecasting

Box 2 outlines some of the key issues raised. A number of respondents pointed out that it is more difficult to plan for the long term, because the evidence base for longer-term interventions tends to be weaker. There is the tendency to favour interventions supported by RCT-level evidence, which are generally short term. This relates to evidence around public health interventions, and also to do with the quality of projections around demographic and societal changes. The impression was that current forecasting tools could be rather crude.

"... if you're looking for robust evidence then thus far the gold standard has been RCTs which therefore is almost always short term."

Decision maker – National Level

"Accuracy of predictions – I think about how we used to do it, it was kind of finger in the air stuff! You would draw a spreadsheet...draw a straight line and that would determine the number of beds"

Public Health Consultant – National Level

Some participants noted that longer term planning was fraught with uncertainty – emergent public health challenges may exert an important influence and these are hard to predict.

"3 years time is fairly easy to predict with some certainty but if you go 5 or 10 years down the road one of the biggest drivers might be climate change or global food shortages"

Director of Public Health – Regional Level

One academic suggested that we need to develop capacity in the area of long-term planning at a national level.

"We don't have a long term perspective think tank"

Academic

The lack of public health professional engagement with the commissioning process was raised. It was suggested that this could be improved through training – this would enable public health professionals to advocate for longer term planning.

"I think part of the fault is public health training... I am very keen that we develop capacity and leadership of Directors of Public Health and senior public health staff in the commissioning process...they need to get their hands dirty in commissioning. I mean the standard response must not be "I'll go away and do a needs assessment and come back and tell you the answer in 12 months". It has got to be timely, it has got to be robust, it has got to speak their language"

Public Health Consultant – Regional Level

## Discussion

Many public health decision makers felt that the short-term time constraints imposed by political and commissioning cycles, and service reorganisations can prevent long-term strategy and investment for public health. This qualitative study explored the factors that influence how far ahead decision and policy makers are planning in public health. We used the decision-making process around CHD as an example.

'Focussing upstream' has become a public health mantra [11], but it requires long term planning, and vision. In this study respondents identified significant 'system' constraints that drive short term planning and decision-making. These include the political cycle, and the need for public organisations to show benefits quickly. Likewise the drive in the commissioning cycle [12] to 'balance the books' in the short term was identified as problematic by some respondents. The governmental comprehensive spending review interval has increased from one to three years, in a move to reduce short-termism [13], but policy makers in health still appear to feel constrained. In addition, the perceived frequency of restructuring within the NHS was cited as being particularly disruptive for long term planning. This is in line with previous studies that have explored barriers to evidence based commissioning [5,14]. It is noted that policy-makers largely identified external factors when trying to explain the short-termism in decision-making. This may represent a form of blame diffusion, and it is concerning that public health practitioners perceive that they have little room for manoeuvre in remedying these issues. Since it is unlikely that governmental cycles are likely to change significantly, it is important that public health gets its own house in order. The only theme that emerged relating to internal factors relates to a perceived lack of training and engagement of pubic health professionals with the commissioning process. It is essential that public health practitioners develop the necessary skills to influence commissioning organisations if we are to achieve population health improvements through 'world class commissioning'[3].

The weaknesses of research evidence for long term planning were emphasised. There is a drive to make public policy in the UK more research based [15,16], and Ovretveit advocates an inclusive definition of research, encompassing a range of methodologies[1]. The current biomedical evidence based paradigm in health tends to favour the generation of evidence around shorter-term interventions and treatments that are easy to evaluate in a randomised control trial. Thus policy makers are likely to invest in this type of intervention, if the strength of the evidence is an important criterion for decision-making. Following on from this there is the suggestion that the laudable focus on evidence-based policy making may increase myopia in policy makers, in the perceived absence of research to support longer-term complex interventions. Another issue is that of timely access to research to inform policy. It is important for researchers to develop methodologies and tools that will allow policy makers to rapidly access evidence to advocate for the longer term and complex interventions that many believe are necessary to address health determinants. Such tools exist, for instance the London Health Observatory Inequalities Tool[17], but in general they are not currently being used by decision makers[8]. It is also important that researchers appreciate the constraints of 'real-life' decision-making. In addition, closer collaboration between researchers and policy-makers is advocated, to facilitate a shared understanding. For example, a key component of the development of the IMPACT decision support tool has been consultation with policy-makers and end-users.

A few studies have highlighted similar issues. A systematic review of decision makers' attitudes to evidence identified the lack of 'timely' access to research as a significant barrier to evidence based decision making [18], and the limited research base for prevention has been highlighted in other studies in the NHS [19]. In a qualitative study Hunter and Marks use semi-stuctured interviews to explore incentives for decision makers to focus on the wider health agenda, and caution against the wholesale import of an acute sector targets based culture in public health [20]. In another study of policy makers Hunter et al suggest that "For a public health system to be truly effective, several interviewees suggested that policies, targets and interventions ought to be more closely based on the available evidence and information, both at a local and national level" [21].

In our study interviewees seemed familiar with the notion that there is a discrepancy between the public health goals timeframe and political and economical cycles. We have sought the opinions of a wide range of public health professionals involved in making decisions around coronary heart disease. In order to ensure inclusion of individuals from as many as possible of the professional groups involved in the policy and decision making process we specifically asked interviewees to recommend groups or individuals for involvement in the study. Although about half of the original sampling frame did not accept the invitation to take part in the study, the final sample is purposive, in that it reflects the diversity of participants that we set out to interview [22]. Coronary heart disease is one of the most important contributors to mortality and morbidity in the UK [23], with well-established organisational frameworks for decision-making. In this respect the CHD decision-making process serves as useful case study, and the issues around timeframes raised here are likely to be equally evident in other areas of decision-making.

This study has various limitations. With regard to reflexivity, the researchers who conducted the interviews and undertook the analysis were from a public health background, with a bias towards a population health perspective – as with all qualitative research it is possible that this could bias the interpretation of data. Although we have cast the net widely in terms of participants, the sample may not represent the full spectrum of views with regard to the decision making process in public health more generally. For instance, local authority staff are represented only by jointly appointed Directors of Public Health, and we were not able to interview Chief Executives. We acknowledge the importance of the views of local government, and their central role in affecting the broader determinants of health. In addition it is difficult to accurately represent the views of subgroups within the sample, and explore differences between groups. This is because of the large number of professional groups and organisational levels at which decisions are made around CHD. We therefore identified common themes that arose across the whole sample. There is scope for more research in this area to map out the differing needs of professional groups involved in the decision making process. The findings in this paper are of particular relevance to decision making around CHD in the UK. We note that respondents in our study highlighted differences in policies and structures between England, Wales and Scotland, and these are explored in a separate paper [24].

## Conclusion

We have identified systematic barriers to long term planning in public health, some of which will be difficult to change. Decision makers should be aware of these factors. Ideally they need to look beyond short-term targets and budget cycles to secure investment for long-term public health.

Improving public health professional's skills in influencing the commissioning process will help. There is also a pressing need to increase the quantity, relevance and accessibility of research for public health decision-making, and to develop tools to support the rapid synthesis of information. This is especially the case around long-term or complex public health interventions designed to influence determinant factors such as dietary interventions in schools, or adjustment to the built environment to facilitate exercise. The IMPACT CHD policy model is being designed with these requirements in mind.

## Competing interests

The authors declare that they have no competing interests.

## Authors' contributions

DTR designed the study, carried out the interviews, analysed the data and drafted the manuscript. BM designed the study, carried out the interviews and analysed the data. FLW participated in its design and coordination of the study, analysed the data and helped to draft the manuscript. MOF participated in the design and coordination of the study, and helped to draft the manuscript. SC conceived of the study, and participated in its design and coordination and helped to draft the manuscript. All authors read and approved the final manuscript.

## Pre-publication history

The pre-publication history for this paper can be accessed here:


